# Challenges in Assessing the Cost-Effectiveness of Newborn Screening: The Example of Congenital Adrenal Hyperplasia

**DOI:** 10.3390/ijns6040082

**Published:** 2020-10-25

**Authors:** Scott D. Grosse, Guy Van Vliet

**Affiliations:** 1 National Center on Birth Defects and Developmental Disabilities, Centers for Disease Control and Prevention, Atlanta, GA 30341, USA; 2 Endocrinology Service and Research Center of the Sainte-Justine Hospital and Department of Pediatrics, University of Montreal, Montreal, QC H3T 1C5, Canada; guy.van-vliet@recherche-ste-justine.qc.ca

**Keywords:** neonatal screening, congenital adrenal hyperplasia, economic evaluation, cost-effectiveness, health policy

## Abstract

Generalizing about the cost-effectiveness of newborn screening (NBS) is difficult due to the heterogeneity of disorders included in NBS panels, along with data limitations. Furthermore, it is unclear to what extent evidence about cost-effectiveness should influence decisions to screen for specific disorders. Screening newborns for congenital adrenal hyperplasia (CAH) due to 21-hydroxylase deficiency can serve as a useful test case, since there is no global consensus on whether CAH should be part of NBS panels. Published and unpublished cost-effectiveness analyses of CAH screening have yielded mixed findings, largely due to differences in methods and data sources for estimating health outcomes and associated costs of early versus late diagnosis as well as between-country differences. Understanding these methodological challenges can help inform future analyses and could also help interested policymakers interpret the results of economic evaluations.

## 1. Introduction

Newborn screening (NBS) has been shown to save lives and healthcare costs [1]. The balance between the costs of screening all newborns, few of whom are affected, and the economic and health benefits, is a concern for some policymakers in deciding whether to add conditions to an existing screening program [2]. Although some jurisdictions use economic assessments to inform decisions on NBS expansion [3], policy decisions often do not require a demonstration of cost-effectiveness, although costs may be considered in some way [4,5,6,7]. For example, the US government does not consider cost-effectiveness when deciding which disorders to recommend for addition to NBS panels [8,9]. Nonetheless, evidence on cost-effectiveness may still influence policy decisions. The purpose of this article is to review economic evaluations of NBS for one disorder, congenital adrenal hyperplasia (CAH), to dig deeply into the factors that can affect calculations of cost-effectiveness in NBS.

Methodological challenges in economic evaluations of NBS include gaps in knowledge of health outcomes and costs of care among individuals with NBS disorders in unscreened population cohorts, confounding of the availability of screening with the availability of effective treatments, and changes over time in the clinical management of conditions, with improved outcomes that may be independent of the availability of screening. The use of outcomes data from historical controls can substantially overstate the health and economic impacts of NBS by confounding screening with improved health outcomes resulting from improved treatments [10]. Available information on treatment costs is often incomplete and inconsistent. Estimates of the proportional reduction in hospitalization costs for children with cystic fibrosis attributable to NBS for cystic fibrosis included in economic evaluations range from 0% to 85% [10]. 

Published economic evaluations have adopted widely varying assumptions about the frequency of adverse health outcomes such as long-term disability or infant/child death in the absence of NBS [6,11,12]. For example, the assumed percentage of children with congenital hypothyroidism detected following NBS who would have otherwise experienced long-term intellectual disability has varied across studies from 4% to 100% [6]. Epidemiologic data, not considered in the preparation of most economic evaluations, showed that 8–28% of children with clinically significant congenital hypothyroidism born prior to NBS were diagnosed with intellectual disability; furthermore, children with subclinical hypothyroidism were not at increased risk of intellectual disability [13].

Other challenges in interpreting economic evaluations of NBS include a lack of standardization in cost-effectiveness methods, which is not unique to NBS. Economic evaluations differ in which costs are included, how costs are estimated, and whether and how costs are adjusted for inflation and time preferences (discounting). Depending on how researchers frame the comparison between screening and its alternative and which assumptions are selected, results may imply that screening is either highly cost-effective or is unlikely to be considered cost-effective. 

The remainder of the introduction provides an overview of CAH epidemiology, screening, treatments, and outcomes. The classic or severe form of congenital adrenal hyperplasia (CAH) due to 21-hydroxylase deficiency is a rare, life-threatening endocrine disorder that is generally found in between 1 in 10,000 and 1 in 20,000 infants. Individuals with a lesser degree of deficiency of the 21-hydroxylase enzyme are classified as having non-classic CAH, which is milder, although it can cause female infertility [14]. Non-classic CAH is usually undiagnosed, with a population prevalence of perhaps 1:500 [15]. 

Classic CAH cases occur along a spectrum that is commonly divided between salt-wasting (SW) and simple-virilizing (SV) subtypes based on the primary clinical presentation, although there is frequent overlap. Based on clinical symptoms, traditionally, roughly 75% of infants with classic CAH were classified as SW-CAH, but upwards of 90% of infants with classic CAH may now be classified as SW-CAH cases based on biochemical measures and genotype [16,17,18]. Infants with classic CAH, not limited to those initially classified as SW-CAH cases, are at risk of acute SW and adrenal crises that, if undetected and untreated, can lead to death, usually in the newborn period. Classic SV-CAH is characterized by virilization of female genitalia due to androgen exposure in utero without SW crises. In the absence of NBS for CAH, genetic females may be assigned male sex until a SW crisis leads to the diagnosis. Discussion with parents about early genital surgery in these cases can be a contentious issue, regardless of whether the diagnosis follows NBS or not. With early diagnosis of classic CAH, glucocorticoid treatment mitigates rapid postnatal growth and progressive virilization in both males and females [15].

In a growing number of countries and regions, classic CAH is detected by NBS programs using an assay that measures the amount of a steroid, 17-hydroxyprogesterone (17-OHP), in dried blood spot specimens (DBS) collected on filter paper cards [19]. However, reference ranges for 17-OHP vary greatly with gestational age and birth weight, as well as acute stress. Because of the very high false-positive rate in first screens among preterm births, single-tier 17-OHP screening has a low positive predictive value (PPV) [20,21,22]. Various methods have been devised to raise the PPV of CAH NBS. Several programs have adopted second-tier screening to reduce the number of infants who need to be recalled for confirmatory testing. Some states conduct 17-OHP fluoroimmuoassay testing on repeat specimens, using extraction to remove cross-reacting steroid sulfates, other states conduct second-tier screening using liquid chromatography tandem mass spectrometry (LC–MS/MS) and still others conduct 17-OHP testing on repeat specimens to reduce the number of infants who need to be recalled for confirmatory testing [15,23,24,25]. A DNA-based second-tier screening test can also be used in place of LC–MS/MS. 

False-negative results also occur, especially with filter paper specimens collected <72 h after birth [26,27,28,29] and especially among infants born preterm, although most appear to be relatively mild cases [30]. In US state screening programs that collect routine second screen specimens at roughly two weeks after birth, as many as 25% of CAH cases are detected only on the second screen, and they are disproportionately milder cases, including both SV-CAH and non-classic CAH [31,32]. Consequently, current CAH NBS protocols have limitations in both specificity and sensitivity to detect all infants with classic CAH who could benefit from early treatment [33]. A DNA-based second-tier test using a panel of selected CYP21A2 gene variants retrospectively piloted in Minnesota in combination with lower 17-OHP cutoffs was found to reduce both false negatives and false positives relative to the existing two-tier screening protocol using LC–MS/MS [34].

The primary public health rationale for CAH screening is the avoidance of mortality and acute morbidity from adrenal or SW crises in neonates with classic CAH [35]. Such crises typically occur in the second or third week of life, sometimes prior to the reporting of screening results. Rapid collection, transport, and processing of specimens and rapid reporting of markedly abnormal results can allow for early onset of treatment prior to the emergence of severe symptoms and can reduce the need for, or length of, neonatal hospitalizations [36]. In one pilot screening study, six unscreened males with SW-CAH were all hospitalized with adrenal or SW crises, compared with none of five screened males with SW-CAH [37]; another study found 0/17 and 10/40 frequencies of hospitalization in screened and unscreened infants with CAH [17].

Even in the absence of screening, improvements over time in clinical recognition and management have greatly reduced rates of death among infants with CAH. For example, the American Academy of Pediatrics cited a rate of 11.3% mortality related to SW crises among infants diagnosed with SW-CAH in Hungary during 1969–1998 [38]. That overall average masked an 80% decline from a mortality rate of 22.2% during 1969–1983 to 4.5% during 1983–1998 [39]. Grosse and Van Vliet calculated an average of 1.5% recognized mortality among infants with SW-CAH in defined birth cohorts in high-income countries, with a confidence interval of 0.2–5.3% [40]. A few recent studies have reported no known deaths among unscreened cases, which can reflect both small numbers of cases and non-ascertainment of deaths [17,37,41]. Neonatal deaths due to CAH can occur even with screening [42,43]. In addition, deaths from CAH can occur later in childhood despite prescription of glucocorticoid treatment [44,45].

In addition to deaths attributed to CAH, deaths may occur among infants with undiagnosed CAH, which can be assessed through post-mortem screening studies of stored DBS specimens. One study of a sample of 242 specimens for sudden infant deaths, out of 2 million births in Austria and the Czech Republic, found three probable cases of CAH [46]. A second study of stored DBS from 1198 unscreened English infants who had died of any cause at 5 days to 6 months of age, out of 600,000 births, found no CAH cases [47]. Pooling the results from the two studies, the implied frequency of death attributable to SW-CAH in unscreened cohorts with SW-CAH was 2.4%, for a total infant mortality rate of perhaps 4% among those with SW-CAH without NBS. 

Given the low rate of infant deaths from CAH compared to historical figures, increased attention is being paid to the potential long-term health benefits of NBS [16]. Children with classic CAH who are not diagnosed clinically as infants may be at risk of health problems secondary to long-term excess androgen production [48]. In particular, accelerated bone maturation and sexual precocity in both males and females due to a delay in the initiation of medical management can incur costly treatments [16]. On the other hand, there is no consistently reported evidence of worse health-related quality of life for cohorts of adults with CAH who were not screened as infants [49]. One study found that Finnish adults with classical CAH reported similar or better health-related quality of life than the general population [50]; German studies reported similar outcomes or slight differences [51,52]; and studies from the United Kingdom and Norway found worse self-rated health [53,54]. 

Specific health issues experienced by adults with CAH might be unrelated to late age at diagnosis and may reflect under or overtreatment. For example, a Swedish study found that both screened and unscreened cohorts of adults with CAH were at increased risk of psychiatric morbidity and substance use disorders [55,56]. Some health problems may be due to long-term, adverse effects of CAH treatments. Reduced cognitive performance in a subset of Swedish children and adolescents with CAH was attributed to treatment in utero with dexamethasone [57]. Long-term treatment with glucocorticoids for various conditions has been shown to result in reduced bone mineral density and increased risk of fractures, in proportion to the cumulative dose [58,59]. 

Acute SW and hypoglycemic episodes that occur in the first years of life among children with classic CAH have sometimes been reported to result in lifelong neurological impairment and intellectual disability [37], although others have found no lasting effects [60]. One study of long-term outcomes among 108 patients with CAH born in Finland during 1980–1995, without NBS, reported that two subjects experienced permanent neurological deficits secondary to severe acute cortisol deficiency after the neonatal period, one of whom was diagnosed with epilepsy and the other with mild intellectual disability [50]. Donaldson et al. reported a high frequency of learning disabilities in English children with CAH, especially among those who had experienced hypoglycemia [61]. However, other studies from high-income countries have consistently reported no excess in cases of intellectual disability among unscreened cohorts of children and adolescents with CAH, relative to either the general population or unaffected siblings [62,63,64,65]. In a single-center study of unscreened Canadian children with CAH born between 1981 and 2001, school progress was not significantly different from that in the general population [66]. A national, all-ages registry of Swedish subjects with CAH also found no difference in diagnoses of intellectual disability, either for those born before or after the implementation of NBS in 1986 [55,56]. Females with both types of classic CAH were less likely to complete the primary school curriculum, which researchers attributed to social or psychological issues secondary to prenatal androgen exposure, not hypoglycemia, since males had no difference in school progress [67]. 

## 2. Review of Previous Estimates

Cost-effectiveness analyses (CEAs) compare costs and outcomes of different strategies, which generally include the status quo. A partial CEA reports changes in costs along with associated intermediate outcomes, such as the number of cases detected, but does not assess health status. In a full CEA, analysts separately calculate monetary costs and health outcomes, such as deaths averted or life-years (LYs) saved. If net health outcomes and net costs are both positive, i.e., better health and higher costs with the intervention, an incremental cost-effectiveness ratio (ICER) is calculated by dividing net costs between the two strategies by the difference in health outcomes. If, instead, health is improved but costs are lower with the intervention, the intervention is said to dominate the comparator, i.e., the intervention is “cost-saving.” In that scenario, no ICER needs to be calculated since it would not be meaningful.

Health outcomes can be expressed in either natural units, e.g., deaths or cases of symptomatic disease averted, or a summary measure of health. The most common summary measure is the quality-adjusted life-year (QALY), which assesses the loss of healthy LYs resulting from both morbidity and premature mortality. The number of QALYs is calculated by multiplying the number of years lived in a health state by a preference-based quality of life measure on a scale from 0 (death) to 1 (perfect health), also called a health-state “utility” value. A CEA that uses QALYs is also referred to as a cost-utility analysis (CUA). Although experts recommend that CEAs use QALYs whenever feasible, it can be challenging to estimate health state utilities for children, which are sometimes extrapolated from adult samples or use adults as proxy respondents [68]. Published utility estimates may be lacking for rare diseases, and some analysts select “off-the-shelf” health state values for other diseases [69]. An alternative is to model reductions in the frequencies of neurodevelopmental sequelae, such as cerebral palsy and hearing loss, and use published catalogues of utility weights stratified by severity of impairments [70]. 

Economic assessments include different types of costs depending on the study perspective. Health economists usually follow either a healthcare perspective that only includes formal healthcare costs or a societal perspective that also includes non-medical costs, such as out-of-pocket costs on transport, special education services for children with learning disabilities, and the “indirect” costs of time spent seeking or providing care, both formal and informal care, and productivity lost due to premature death or disability. The societal perspective is regarded as particularly appropriate for public health policies and programs, so that decision makers can take into account health and non-health interventions and outcomes that affect societal well-being [71]. A narrower perspective can also be taken that focuses on budgetary costs to publicly funded programs. 

If an intervention is both more effective and more costly, i.e., has a positive ICER, decision makers can decide if they consider the intervention to yield good value for money relative to other types of interventions. Analysts often compare their estimates to a threshold or benchmark value, such as USD 50,000 per discounted QALY gained, to inform decision makers [72,73]. Globally, the World Health Organization has suggested a range of one to three times gross domestic product (GDP) per capita per disability-adjusted LY, although relatively few interventions are not classified as cost-effective on that basis [74]. Although LYs are commonly treated as interchangeable with QALYs, since average utility weights are less than 1.0 and decrease with advancing age, the cost per QALY gained may be 15–20% larger than the cost per LY gained [75]. 

The present review summarizes and critiques previous estimates of the costs and cost-effectiveness of NBS for CAH. These estimates include two partial economic evaluations conducted in the United States (US) during 1998–1999, which provided information that could be used to inform CEAs [63,76]. Two CEAs published in 2006 and 2009 yielded divergent estimates of cost-effectiveness [77,78]. In addition, we review two assessments of the treatment costs and outcomes of CAH by researchers in the province of British Columbia, Canada [17], and in the state of Sao Paulo, Brazil [16]; both studies were favorable to NBS for CAH. The Canadian manuscript combined an assessment of costs and outcomes in screened and unscreened cohorts (primary study) with a modeling study of costs and avoided hospitalizations that authors referred to as a “cost efficacy” analysis [17]. The Brazilian study split the primary study and modeling study between two manuscripts; only the primary study has so far been published [16]. 

Another unpublished Australian CEA of NBS for CAH is not in this section but is addressed in the Discussion. This 2017 CEA was prepared as part of a multifaceted decision process by the Standing Committee on Screening of the Australian Government Department of Health to recommend the adoption of NBS for CAH, a recommendation that was subsequently endorsed by the Australian Health Ministers’ Advisory Council. The 2019 summary assessment noted that the CEA estimated the cost of NBS as AUD 2.14 per infant screened and the ICER as AUD 73,504 per QALY, which was slightly higher than GDP per capita [79]. The report concluded, “newborn screening for classic CAH could be considered to be cost effective”.

### 2.1. Cost and Cost-Effectiveness Estimates in the United States

In 1998, Brosnan et al. published a cost accounting analysis of NBS for CAH in the state of Texas during 1994 [76]. That article was complemented by a 1999 study by Brosnan et al. that compared hospitalization patterns among infants with CAH born in Texas and in two adjoining states, Arkansas and Oklahoma, which did not at the time screen for CAH [63]. The incremental cost analysis calculated the variable costs of specimen testing, reporting and short-term follow-up, and diagnostic evaluation, as well as the incremental cost per diagnosed case [76]. Most infants in the 1998 study required hospitalization, a median of 6 days for the eight infants diagnosed based on screening results and 9 days for seven infants detected clinically, prior to screening results. The 1999 study reported that male infants in the screened Texan cohort had significantly earlier age at diagnosis (12 vs. 26 days) and fewer hospitalization days (median length of stay 8.0 vs. 15.5 days), but little difference in the proportion hospitalized (90% vs. 100%) [63]. 

In 2005, the first nationally recommended NBS panel in the US was adopted, and it included classic CAH; ten US states started screening for the disorder after the recommendation [24]. The same 2006 journal supplement contained the reports supporting the screening panel [80] together with a commissioned economic evaluation of disorders on that panel, including CAH [77]. Carroll and Downs assessed the cost-effectiveness of screening for seven individual disorders, one of which was CAH. The authors calculated that screening for five disorders would be cost saving and asserted that their assumptions were “pessimistic,” i.e., conservative [77]. However, a number of the assumptions in that study for conditions, such as congenital hypothyroidism and phenylketonuria, appear to have been insufficiently conservative in comparison with the best available evidence [10,11]. Carroll and Downs calculated an ICER in 2004 US dollars of USD 20,357 per QALY gained for CAH NBS compared with no screening. 

Yoo and Grosse reported a “deterministic” ICER of USD 292,000 per LY in 2005 US dollars and a “probabilistic” ICER of USD 255,000 per LY gained [78]. However, a 2018 erratum noted that those results did not accurately reflect the assumptions in the article [78]. Mr. Orban Holdgate, who co-authored the erratum with Dr. Grosse, discovered that the original model incorrectly applied the 80% reduction in mortality among infants with SW-CAH to the subset of infants with SW-CAH who were not clinically diagnosed prior to NBS. A corrected estimate of the deterministic ICER, maintaining all assumptions in the original article, was USD 128,000 per LY saved in 2005 US dollars; the adjustments accounted for 60% of the gap between the published USD 292,000 ICER estimate and the roughly USD 20,000 Carroll and Downs ICER estimate. Dr. Grosse did not have access to the probabilistic CEA model developed by Dr. Yoo.

The primary explanation for the difference in ICER estimates between the Carroll and Downs study and the corrected Yoo and Grosse estimates was different assumptions of the mortality rate in the absence of NBS; both studies assumed an 80% lower mortality rate with NBS (Table 1). Carroll and Downs assumed a 10% mortality rate in untreated classic CAH [77]. Since CAH-related neonatal mortality is specific to SW-CAH, which accounted for approximately 75% of classic CAH cases, the implied pre-NBS mortality rate for those with SW-CAH in the Carroll–Downs analysis was 13.3%. Yoo and Grosse assumed 4.2% mortality among unscreened infants with SW-CAH [78]. Cost estimates contributed little to the difference in ICER estimates. Both studies assumed NBS for CAH cost a little over USD 6 per infant and that hospital costs would be lower with NBS by roughly USD 3000 per infant with SW-CAH. Finally, Carroll and Downs reported USD 10,000 lifetime treatment cost, although it is unclear how that influenced the ICER estimates.

### 2.2. Economic Estimates in Canada

In their primary study, Fox et al. reported costs of treatment and outcomes for two cohorts of pediatric patients with classic CAH, born in the Canadian province of British Columbia (BC) [17]. One was an unscreened cohort of 40 persons born during 1988–2008 who were referred to BC Children’s Hospital for treatment [17]. Although the authors did not indicate the fraction of patients with CAH referred to BC Children’s Hospital, applying the CAH frequency from NBS data to the number of births in BC during 1988–2008, roughly 80% of infants born with CAH during that period were referred. The 10 missing cases could have included deaths without diagnosis as well as children who did not require tertiary care. In contrast, the screened cohort consisted of all 17 infants detected with CAH in BC from the date NBS started in November 2010 through February 2018.

Fox et al. reported the median age at diagnosis as 5 days in the unscreened cohort and 6 days in the screened cohort [17]. More crucially, among unscreened and screened males, the median ages at diagnosis were 14 days and 5.5 days, respectively, and 38% of the unscreened infants had a salt-wasting crisis, versus none of the screened males. Among infants of both sexes, 85% of unscreened infants and 29% of screened infants were hospitalized, among whom the mean length of stay was 11.1 days and 4.8 days, respectively, a difference of 6.3 days. The average number of neonatal/pediatric intensive care unit (ICU) days for screened and unscreened infants was comparable, 0.65 and 0.70 days, respectively. Average hospital costs were twice as high in the unscreened cohort, CAD 33,770 (2018 Canadian dollars) vs. CAD 17,726, a net difference of negative CAD 16,044. The cost of hospital transfers averaged CAD 3075 and CAD 2286, respectively.

The overall cost of the NBS program was CAD 73,690.51 per case, or CAD 3.78 per infant screened using a birth prevalence of 1 in 19,510 (Table 1). The unit costs of screening and follow-up were reported to be CAD 2.7 per specimen for first-tier screening, CAD 19 for second-tier screening, and CAD 91.1 for testing with a second specimen. The modeling study combined net hospital costs with screening and follow-up costs. The modeling study presented two incremental cost analyses, one for 2-tier testing and one for first-tier testing. 

Fox et al. reported an incremental cost per hospital day avoided of CAD 4746 for the base-case analysis [17]. That ICER is difficult to interpret as a measure of NBS cost-effectiveness for several reasons. First, it does not provide an adequate basis of comparison with cost-effectiveness findings for other interventions; we found only two peer-reviewed CEAs that reported the cost per hospital day avoided as a primary outcome measure, both assessments of schizophrenia treatments. Second, there are no estimates of decision makers’ willingness to pay to avoid hospital days. Third, the denominator may be problematic as hospital days effectively entered the ICER in two places, directly in the denominator and indirectly in the cost numerator as the main driver of costs of care. Finally, the calculation by Fox et al. of the cost numerator for the ICER is uncertain; the authors did not report their calculations and did not provide enough information for readers to replicate their estimates. In the modeling study, the mean number of avoided hospital days was assumed to be 6.3 days, which is the difference in mean length of stay among infants with CAH who were hospitalized in the primary study. Multiplying 6.3 days per infant with CAH by CAD 4746 yields a total incremental cost of CAD 29,900 per case. Subtracting the avoided cost of hospitalization (CAD 16,044) yields an incremental screening cost of CAD 45,944 per case, which is 38% lower than the NBS cost of CAD 73,690.51 per case reported in the primary study.

### 2.3. Economic Estimates in Brazil

Researchers at the Medical Faculty of the University of Sao Paulo have a two-phase process to evaluate the cost-effectiveness of CAH screening in the Brazilian state of Sao Paulo, which initiated universal NBS for CAH in November 2013 [28]. The two phases consist of a primary study of data and outcomes in an unscreened cohort in which inferences about health and economic benefits are drawn by assuming improved outcomes with NBS. The second phase is a modeling CEA study. In the primary study, Miranda et al. analyzed medical records of 195 children with CAH treated at their hospital during 1980–2016, 54 (28%) of whom were male and 105 (54%) of whom had SW-CAH [16]. Those numbers reflect an under-ascertainment of males and of children with SW-CAH, particularly in older cohorts. In a corrected Table 2 submitted by Miranda et al. as an erratum, the percent males among diagnosed CAH cases for three birth cohorts, <1989, 1990–1999, and >1999, were 25%, 29%, and 39%, and the percent with SW-CAH were 43%, 62%, and 70%, respectively [16]. The distribution of CAH cases by those characteristics for the third birth cohort was closer to what one would expect based on data from other countries, but due to small numbers, the authors chose to combine the second and third cohorts, 64% of whom had SW-CAH. They conclude that, prior to the implementation of NBS for CAH in Sao Paulo, 10–26% of infants with CAH died. That range was calculated as the difference between 64% in the unscreened cohort and an expected 75% to 90% percentage of SW-CAH cases with screening. However, the 90% estimate reflects children with biochemical evidence of SW-CAH, not clinical diagnoses, which are the relevant comparison for clinical diagnoses of SW-CAH in unscreened cohorts. For the most recent birth cohort, the mortality rate calculated as the deficit in SW-CAH cases relative to clinical diagnoses of SW-CAH in screened cohorts was 5%, i.e., 75% minus 70%. Miranda et al. multiplied their hypothesized numbers of deaths from unascertained SW-CAH cases by GDP per capita for Brazil and by 34.5 years of working life and discounted costs in future years by 5% annually.

Miranda et al. reported that almost all patients experienced dehydration (85%) and hospitalization (91%), regardless of birth cohort [28]. Despite lengthy hospitalizations, with stays of 30–47 days and over one-third requiring intensive care, the mean cost per SW-CAH case was reported to be a surprisingly low USD 1087 (2016 US dollars), reflecting low medical prices reported in Brazil. In contrast, the relatively few SW-CAH cases with neurological impairments, either cerebral palsy (*n* = 2) or intellectual disability (*n* = 7), were projected to incur very high costs, which were said to average USD 85,156, comprising the majority of all treatment costs attributed to CAH in their cost analysis [16]. That estimate was generated by multiplying the numbers of cases by US estimates of lifetime costs for those two disabling conditions of approximately USD 1 million per child in 2003 [82]. However, roughly 80% of that amount was the projected loss of economic productivity from premature mortality and work disability due to intellectual disability or cerebral palsy, not the direct costs of treatment. Furthermore, Miranda et al. made no adjustment for differences in prices and average earnings between Brazil and the US [16]. Finally, Miranda et al. tracked services and costs associated with management of complications of SV-CAH for up to 19 years after birth, and they estimated a total cost of almost USD 6000 per child with SV-CAH [16]. The authors suggested that “*the frequent use of expensive therapies such as GnRHa and GH in our cohort…could have been avoided with an earlier diagnosis.*”.

## 3. Discussion

Multiple authors have called attention to the conflicting cost-effectiveness findings of the two US CEAs from 2006–2009 and suggested that further research could help bring clarity [7,15,16,17,19]. What has been learned in the past decade? First, the estimates from the second US CEA were substantially overstated; the corrected ICER estimate of USD 128,000 per LY [78] was two-fifths of the original estimate of USD 292,000 per LY in 2005 US dollars [78]. That illustrates the importance of full transparency in cost-effectiveness modeling. Authors ideally should report all calculations to reviewers and also make them available to readers. Independent peer review of cost-effectiveness models, not just manuscripts, can help avoid errors in published results [83]. Finally, post-publication peer review of economic assessments can contribute to an improved understanding of estimates, analogous to post-marketing surveillance of pharmaceuticals. 

The assessment of cost-effectiveness is inherently subjective, contingent on available resources and the preferences and priorities of decision makers. Although the corrected ICER of USD 128,000 per LY in 2005 US dollars from Yoo and Grosse still exceeded the commonly cited range of USD 50,000 to USD 100,000 per QALY or LY thresholds [7,72,73], a broader range might be considered appropriate for interventions targeting a rare disorder such as CAH. The upper-bound WHO-endorsed cost-effectiveness threshold of three times per capita GDP for the USA was roughly USD 150,000 per DALY/QALY as of 2009. Taking into account the fact that one LY may be equivalent to 0.8–0.9 QALYs, CAH NBS could have been considered cost-effective on that basis. Moreover, decision makers in some countries have been reported to consider the acceptable upper-bound ICER for treatments for rare diseases to be as much as three times greater than the usual upper-bound ICER threshold, e.g., an upper bound of roughly USD 500,000 per QALY [84,85]. If that same benchmark was applied to the combination of screening and treatments for rare disorders [86,87], CAH NBS could be more readily justified as cost-effective. 

Most of the residual difference in the two US ICER estimates, i.e., USD 128,000 and USD 20,000 in 2004 or 2005 US dollars, appears to be due to an unrealistically high estimate of CAH-attributable infant mortality rate for a high-income country assumed by Carroll and Downs, which was three times the rate assumed by Yoo and Grosse for unscreened infants with SW-CAH born in high-income countries (Table 1). Fox et al. in their modeling study assumed no excess deaths from CAH in either screened or unscreened cohorts, based on their historical cohort of treated patients born in British Columbia prior to the implementation of CAH NBS [17]. However, given the small numbers of cases, the rarity of neonatal death in SW-CAH in high-income countries, and potential underascertainment of deaths, it should not be surprising that no deaths were reported. In contrast, the unpublished Australian CEA took a similar approach to Yoo and Grosse, using mortality estimates from high-income countries to calculate gains in life-years from CAH NBS. 

Similarly, the other parameters in the Yoo and Grosse model merit reexamination in light of evidence that the numbers of crises, lengthy hospitalizations, and deaths in infancy associated with SW-CAH have fallen over time with improvements in clinical recognition and management of CAH, with or without NBS. One of the most important contextual factors in assessing the benefits of NBS for CAH is the timing of diagnosis following NBS. Faster reporting of abnormal screening results and prompt initiation of treatment can greatly reduce the occurrence of poor outcomes in screened cohorts. For example, in New Zealand during the first decade of screening for CAH (1984–1993), many infants were not diagnosed and treated until after 10 days of life, at which point some were in crisis [88]. Subsequently, timely reporting was achieved, and no neonatal SW crises were reported during 1994–2013 [89]. Some programs have likewise reported avoidance of SW crises through timely reporting of NBS results [17,23], whereas others report that even timely reporting does not eliminate crises [35].

The majority of economic evaluations of CAH NBS modeled only short-term improvements in health outcomes among infants with SW-CAH. The exceptions are the Australian and Brazilian modeling studies, which also modeled a reduction in long-term neurological sequelae among children with SW-CAH detected through NBS. The Brazilian primary study reported neurological impairment in 9 of 105 (8.6%) unscreened children with SW-CAH [16]. The unpublished Australian CEA assumed that SW-CAH is clinically analogous to medium chain acyl-CoA dehydrogenase (MCAD) deficiency in risk of crises that result in neurological impairment and disability. The Australian CEA projected gains in QALYs and avoided costs from prevention of disability, informed by a French CEA of MCAD deficiency NBS that assumed 6.7% of children with MCAD deficiency in the absence of screening develop permanent neurological damage and loss in health utility [90]. In contrast, an Australian CEA of MCAD deficiency NBS only modeled gains in LYs [91]. Although mild neurological impairment (developmental delay at age 4 years or need for special education services at age 6 years) was observed among unscreened Australian children with MCAD deficiency born 1994–1998, no unscreened children born 1998–2002 were found to be similarly affected [92,93].

The risk of neurological impairment secondary to acute crises experienced by young children with SW-CAH may be lower than some experts think. A Finnish study reported that 1.9% of an unscreened cohort of children with SW-CAH experienced disability following crises [50], which is less than one-third the frequency assumed in the French modeling study for MCAD deficiency. Other studies, with the exception of an older study from England [61], have not documented disability among children following SW-CAH crises. A German study documented crises during the first 6 months of life in a cohort of children with CAH detected by NBS and found that 28% had salt-losing or hypoglycemic episodes and 18% experienced seizures, but developmental status at ages 4–6 years was normal in all children and did not differ between those who had crises or seizures and those that did not [60]. Multiple studies, including a large-scale Swedish registry linked to a national patient database, have found no indication of intellectual disability due to CAH complications, with or without screening [55,56]. Thus, the available evidence does not support avoidance of long-term neurological disability as a putative benefit of CAH NBS in high-income countries.

Both economic and epidemiologic inputs in models of economic costs and health outcomes can be influential in cost-effectiveness estimates of NBS both in general as well as for CAH. Although a lower cost of NBS makes it more likely to be considered cost-effective, epidemiologic estimates of the occurrence of acute morbidity, mortality, and long-term outcomes in unscreened and screened cohorts are commonly the most influential determinants of cost-effectiveness estimates in NBS [10,11]. That is logical, since without effectiveness in terms of improved health, an intervention cannot be cost-effective.

The cost of NBS includes the costs of both screening and diagnostic assessments as well as short-term follow-up. The cost of screening per se is a function of both the unit cost of screening tests and the likelihood an infant is recalled for repeat specimen collection and repeat testing. Notably, the Australian and Canadian economic evaluations both assumed a unit cost of roughly USD 2 in 2018 prices for 17-OHP screening, compared with roughly USD 5 in the two US CEAs. In general, the cost of follow-up testing is less influential than the cost of initial screening. Despite a more than 50 times higher unit cost relative to 17-OHP testing, Yoo and Grosse estimated that follow-up and diagnostic testing accounted for just 9% of total costs of NBS [78].

The use of second-tier screens might improve the cost-effectiveness of CAH NBS, depending on the frequency and cost of confirmatory testing and the magnitude of reduction in false-positives. One group in 2004 argued that the use of LC–MS/MS could reduce total screening costs [94], but that analysis assumed an extremely high cost of follow-up testing, USD 848, four times the estimate in the CEA by Yoo and Grosse [78]. Moreover, the observed reduction in numbers of infants referred by use of LC–MS/MS was less than had been predicted [25]. Alternatives to second-tier screening that reduce the number of infants referred for confirmatory testing include repeat screening of preterm infants who screen positive and adjustment of screening cutoffs for birth weight, gestational age, and age at sample collection. No formal CEA modeling of second-tier CAH screening has appeared to date. In contrast, for more than two decades, economic evaluations of cystic fibrosis NBS have regularly modeled the relative cost-effectiveness of various screening algorithms employing second-tier or even third-tier screening tests [10,95,96].

CEAs of NBS for any disorder face multiple challenges. The most important is the availability of data required to reliably quantify the impact of screening on health outcomes relative to unscreened cohorts. Two types of comparisons can be made using observational data on reported cases: concurrent data from similar jurisdictions with and without screening and historical controls born prior to NBS implementation [10,12]. A critical limitation of both concurrent and historical controls is ascertainment bias. One type of ascertainment bias can occur when an affected but undiagnosed child dies from a crisis without undergoing autopsy, or the cause of death is either unrecognized or not reported to authorities. Another type of ascertainment bias happens when a person is mildly affected and not referred for a diagnostic evaluation. In addition to ascertainment bias, referral bias can occur if data are collected by an academic medical center rather than as population-based surveillance. One way to test for ascertainment bias is to conduct retrospective testing of stored DBS collected for unscreened cohorts. For example, investigators in England tested over 100,000 stored DBS specimens and eight children with MCAD deficiency, of whom six (75%) were symptomatic but only four experienced a metabolic crisis, one of whom had died as a result [97]. Previous reports based on clinically referred cases had reported substantially greater proportions of infants with MCAD deficiency with fatal crises [11,12,98].

Three studies reported short-term outcomes of pilot screening for CAH in the US [63], the Netherlands [41], and Australia [37] in comparison with concurrent surveillance data in regions that did not screen for CAH. Historical (pre-post screening) comparisons have been reported from Sweden [81], Brazil [16], and Canada [17]. These comparisons cannot control for changes in clinical management, which can greatly alter outcomes over time, both for CAH and other conditions, such as MCAD deficiency. If jurisdictions that are early adopters of screening for a condition also have higher standards of clinical recognition and management, concurrent comparisons can be subject to similar bias. Two retrospective screening studies of stored DBS specimens for CAH have been published, although only for infant deaths [46,47]. For CAH NBS, a fundamental constraint is the low prevalence of classic CAH. In order to have sufficient case numbers to conduct statistical tests of differences in non-fatal outcomes, specimens for millions of births with and without screening would likely be required, which is not practical.

Grosse and Van Vliet in their 2007 review identified ascertainment and referral bias as threats to study validity for estimates of mortality in unscreened cohorts with CAH [40]. The recently published Brazilian and Canadian primary studies both appear to have been subject to ascertainment and referral biases. In the Canadian study, as noted above, up to 20% of births with classic CAH were apparently not included in the unscreened cohort. If excluded cases were either relatively mild or resulted in sudden death without hospitalization, estimates of hospitalization and costs for the recognized cohort could have been upwardly biased. The Brazilian study did not provide information on the percentage of CAH cases born in the state of Sao Paulo who were referred to the study hospital, which was likely much smaller than in British Columbia given the large population with multiple metropolitan areas and hospitals. Miranda et al. had no access to data on patients with CAH seen at other centers in the state [16]. Therefore, in the Sao Paulo study sample, it is not possible to rule out referral bias as contributing to the frequency of neurological impairments among subjects with CAH. 

Another challenge for NBS CEAs in general is obtaining information on decrements in health-state utilities associated with late-diagnosed disorders in order to estimate QALY gains from reductions in morbidity and disability. CEAs of NBS for metabolic disorders have used a wide range of assumptions about health-state utilities associated with neurological impairments that can yield inconsistent estimates of QALY gains [70]. Some CEAs for specific disorders have applied published utility estimates for other disorders. For example, CEAs of NBS for severe combined immunodeficiency (SCID) have applied utility weights for conditions such as cystic fibrosis, MCAD deficiency, and sickle cell disease [69] or leukemia [99]. In general, though, the frequencies of health states associated with utility decrements may be more influential than the utility weights assigned to those health states. 

Two studies estimated QALY gains from CAH NBS despite a lack of CAH-specific information. First, Carroll and Downs reported ICERs with QALYs as the denominator [77], but since the study assumed no reduction in health-related quality of life for individuals with CAH, the actual denominator was LYs rather than QALYs. Second, an unpublished CEA in Australia modeled QALYs from glucocorticoid treatment, unrelated to age at diagnosis, and from neurological damage secondary to episodes of severe cortisol deficiency. The authors used estimates of health-state utility values from a French modeling study of MCAD deficiency NBS, which reported weights of 0.89 for mild sequelae and 0.76 for severe sequelae of MCAD deficiency [90]. The French study cited a Finnish modeling study as their source [100]. Coincidentally, the latter study included CAH in its analysis of a proposed expanded NBS panel in Finland but did not report cost-effectiveness estimates by individual disorder. 

## 4. Conclusions

The cost-effectiveness of CAH NBS is likely to be context specific. In middle-income countries, such as Brazil, the potential benefit from avoided deaths and impairments may be substantially larger than in high-income countries, but difficult to quantify. In higher-income countries, increased accuracy of NBS practices, including the use of cutoffs adjusted for infant characteristics, the timing of specimen collection, and multi-tier algorithms, can eliminate most false-positive screening results and may improve cost-effectiveness. Timely screening and reporting can greatly reduce the burden to families, hospitals, primary care physicians, and pediatric endocrinologists, by allowing for the initiation of treatment prior to the appearance of serious symptoms. Despite recent attempts to quantify putative long-term outcomes, the main quantifiable benefits of CAH NBS continue to be the reductions in fatalities and acute neonatal morbidity with resultant hospitalizations that have long been modeled in CEAs of CAH NBS. 

It is unclear to what extent cost-effectiveness estimates influence decisions to add disorders, such as CAH, to NBS panels. Since NBS for phenylketonuria was introduced in the early 1960s, the adoption or expansion of NBS has mostly been driven by technological capability, advocacy, and medical opinion rather than through a rigorous evidence-based review process [7,101]. Although advocates may advance economic arguments to screen for a disorder, policy decisions appear to be influenced more by qualitative assessments of the value of prompt identification and early treatment than by quantitative assessments [10,102]. For example, the 2005 recommendation of a uniform screening panel in the US that included CAH was intended to achieve uniformity by endorsing screening for disorders already screened for by most states and cost-effectiveness was not a criterion [6,80]. Few jurisdictions worldwide have formal procedures for the inclusion of cost-effectiveness in NBS policy processes [3,4,5]. Even when such procedures are in place, other considerations may trump cost-effectiveness findings, and it can be challenging to determine how specific decisions were reached [103]. Although Australia commissioned a CEA as part of the decision in 2017 to recommend the implementation of CAH NBS nationally, neither the CEA nor the report explaining how decision criteria were applied to make the recommendation were released. Furthermore, in a federal system, such as Australia, Belgium, Brazil, Canada, and the US, states or regions may decide whether or when to implement CAH NBS. 

## Figures and Tables

**Table 1:** Characteristics, outcomes, and assumptions of published cost-effectiveness analyses (CEAs) of newborn screening (NBS) for congenital adrenal hyperplasia (CAH).

Study and Perspective	Cost-Effectiveness Ratio	Cost of Initial Screening and Confirmatory Testing per Infant Screened	Birth Prevalence of CAH (% with SW-CAH)	SW-CAH Mortality	Excess Hospital Cost for Infants with CAH in Absence of NBS	Other Costs
Carroll and Downs 2006 [77] “Societal” ^1^	USD 20,357 per QALY ^2^ (2004 USD)	USD 3.63 for 17-OHP testing, USS 2.40 for confirmatory testing ^3^ (2004 USD)	1 in 20,400 (SW-CAH share not reported)	13.3% ^4^ without NBS, 2.7% with NBS (80% reduction)	USD 2966 per infant with SW-CAH ^5^ (2004 USD)	USD US10,000 Cost of caring for disease (expert opinion) (2004 USD)
Yoo and Grosse 2009, corrected [78] Healthcare sector	USD 128,000 per LY ^6^ (2005 USD)	USD 4.15 for 17-OHP testing, USD 2.16 for confirmatory testing ^7^ (2005 USD)	1 in 17,800 (75% SW-CAH)	4.2% ^8^ without NBS, 0.8% with NBS (80% reduction)	USD 3000 per infants with SW-CAH ^9^ (2005 USD)	
Fox et al. 2020 [17] Public payer	CAD 4746 per hospital-day avoided ^10^ (2018 CAD)	CAD 3.78 ^11^ CAD 2.70 for 17-OHP testing, CAD 0.99 for second tier screening ^12^ (2018 CAD)	1 in 19,510	No deaths	CAD 16,044 per infant with CAH ^13^ (2018 CAD)	CAD 319 for endocrinology consult per confirmed case

^1^ Carroll and Downs reported following a societal perspective, with a mix of medical and nonmedical costs, but the CAH analysis included only medical costs, consistent with a healthcare sector or system perspective, the same perspective followed by Yoo and Grosse. ^2^ Study reported cost per QALY, but Carroll and Downs actually calculated LYs and called them QALYs. ^3^ USD 300 cost of follow-up testing per false-positive infant multiplied by a 0.8% false-positive rate among screened infants.^4^ Citing expert opinion, study assumed a 10% mortality rate attributable to SW crises for infants born with CAH, equivalent to an excess mortality rate of 13.3% for infants with SW-CAH assuming that 75% of infants with CAH have SW-CAH. ^5^ Study assumed infants with SW-CAH who died (an excess 10.6% mortality without NBS) incur a hospitalization cost of USD 27,809, based on intensive care costs for elderly American decedents. ^6^ Original study incorrectly reported an ICER of USD 292,000 per LY in a base-case deterministic model, as the result of formula errors (see erratum). ^7^ USD 216 per false-positive infant multiplied by a 1.0% false-positive rate.^8^ Sum of 2.2% mortality rate for an unscreened cohort with SW-CAH in Sweden [81] and a roughly 2% rate of unrecognized deaths among infants with SW-CAH in Austria and the Czech Republic [46]. ^9^ Difference between screened and unscreened male infants with SW-CAH of USD 6000, citing two published studies [41,76].^10^ No details of the calculation were reported. ^11^ Total NBS program cost of CAD 73,690.51 per CAH case detected divided by 19,510 equals CAD 3.78. ^12^ Cost of second-tier testing per infant screened was calculated as CAD 19 multiplied by the false positive rate of 5.2% on initial screening. ^13^ Difference between CAD 33,370 average cost for unscreened infants and CAD 17,726 for screened infants (2018 CAD).
